# Body Odours as Lures for Stoats *Mustela erminea*: Captive and Field Trials

**DOI:** 10.3390/ani12030394

**Published:** 2022-02-07

**Authors:** Elaine C. Murphy, Tim Sjoberg, Tom Agnew, Madeline Sutherland, Graeme Andrews, Raine Williams, Jeff Williams, James Ross, B. Kay Clapperton

**Affiliations:** 1 Department of Pest Management and Conservation, P.O. Box 85084, Lincoln University, Christchurch 7647, New Zealand; tim@pestfreebp.org.nz (T.S.); tom@zip.org.nz (T.A.); madeline.sutherland@lincolnuni.ac.nz (M.S.); james.ross@lincoln.ac.nz (J.R.); 2 Department of Conservation, Private Bag 4715, Christchurch 8140, New Zealand; 3 Department of Conservation, P.O. Box 55, St Arnaud 7053, New Zealand; gandrews@doc.govt.nz; 4 Independent researchers, P.O. Box 41, Coromandel 3543, New Zealand; j40gryphon@gmail.com (R.W.); gryphonsubsea@gmail.com (J.W.); 5 Independent researcher, Havelock North 4130, New Zealand; clapperton.lo@xtra.co.nz

**Keywords:** eradication, mustelid, pen trials, predator control, scent, trapping, wildlife management

## Abstract

**Simple Summary:**

The stoat (*Mustela erminea*) is invasive in New Zealand and has a serious impact on native biota. Trapping is the most common technique used to control stoats, but efforts to eradicate them or to improve control efficiency will require a range of different techniques. We examined the use of mustelid body odours as lures to attract stoats to traps or monitoring devices. Stoats were attracted to stoat urine, scats, and bedding, and to ferret (*M. furo*) bedding in captive and field trials. The use of odour lures may be particularly useful when the usual food-based lures are ineffective.

**Abstract:**

Eradication and control methods to limit damage caused to native biota in New Zealand by the stoat (*Mustela erminea*) rely on effective lures for trapping and detection devices, such as cameras. Long-life semiochemical lures have the potential for targeting stoats in situations where food-based lures are of limited success. The attractiveness of body odours of captive stoats was tested in a series of captive animal and extensive field trials to investigate their potential as trapping and monitoring lures. Stoats approached and spent significantly more time sniffing stoat urine and scats and bedding from oestrous female stoats than a non-treatment control. The bedding odours were attractive in both the breeding and the non-breeding season. Stoats also spent significantly more time sniffing oestrous stoat bedding than female ferret bedding, but the ferret odour also produced a significant response by stoats. In the field trials, there were no significant differences between the number of stoats caught with food lures (long-life rabbit or hen eggs) compared with oestrous female or male stoat bedding lures. These results indicate the potential of both stoat bedding odour and the scent of another mustelid species as stoat trapping lures that likely act as a general odour attractant rather than a specific chemical signal of oestrus.

## 1. Introduction

The stoat *(Mustela erminea* L.) is a major threat to New Zealand wildlife [1,2,3,4]. It preys on birds, reptiles and invertebrates in forest and alpine habitats, on shorelines, in riverbeds, and in wetlands [5,6,7,8,9,10,11]. The swimming ability of stoats means that many offshore predator-free islands are within their range [12,13]. In 2016, the ambitious ‘Predator Free 2050’ (PF 2050) programme was announced, which aims to eradicate stoats, rats (*Rattus* spp.) and brushtail possums (*Trichosurus vulpecula* Kerr) from New Zealand by 2050. It seems likely that no single technique will bring about these eradications—a ‘toolbox’ of methods will be required, and research is therefore under way on a wide variety of fronts [14].

Current control techniques for stoats in New Zealand rely mainly on networks of kill traps [4]. These may be run year-round to maintain low predator pressure [15,16,17], only during the bird breeding season, or in response to a stoat population irruption [18,19]. Such trapping programmes require effective long-life lures that will be attractive to all members of the pest population. Brown et al. [4] stated that “the biggest current issue with stoat trapping is that not all stoats enter the traps.” Over time, there may also be a decline in the effectiveness of trapping programmes—long-term trapping operations in one location may select for untrappable stoats that need to be controlled by other techniques, such as poisoning [20].

Food-based lures have long been considered the ‘best practice’ for stoat trapping. Hen eggs and rabbit (*Oryctolagus cuniculus* L.) meat are the current standard lures, and long-life rabbit formulations are available (https://predatorfreenz.org/resources/trapping-best-practice/, accessed on 5 February 2022). However, food-based lures are not always effective at attracting stoats to traps and bait stations [21]. Food preferences will vary among individuals and across sites and times of year [22]. Food lures can also be less effective when ample food supplies are available to stoats [4,23]. Martin [24] found that reduced activity rates of stoats in early summer made tracking tunnel monitoring less effective, so this may be a time when more attractive lures could increase control and monitoring efficacy. Recent innovations such as tunnels that squirt toxins onto body fur [25] and ‘virtual barriers’ [26] could also benefit from effective long-life species-specific lures.

For monitoring and/or detection, current best practice is to bait lines of tracking tunnels with rabbit meat; this provides a coarse index of relative abundance [27]. However, with the uptake of trail cameras (either infrared or thermal) as a monitoring tool and especially as a detection tool in eradications [28,29,30,31], luring helps to entice predators on site to generate a recording [32]. Non-food lures could be effective when attempting to detect individuals within a low-density population, when there is little competition for food. Monitoring and responding to island incursions is such a situation; social odours may be of particular interest to lone invaders [33,34,35] when food-based lures may not be as attractive. 

Fertile female stoats pose the greatest threat to predator-free areas, potentially carrying blastocysts in the uterus year-round and these pregnant females are considered to be the most difficult to catch [36]. A limitation for using rabbit as a trap lure is that while rabbit can be the major component of stoat diet in open habitats [5,7], it is less frequently consumed in podocarp and beech forest habitats [37,38]. Thus, rabbit may not be a learnt food if stoats, like ferrets (*Mustela furo* L.), use olfaction early in life to imprint on prey species [39]. Egg baits also have their limitations. Female stoats consumed them less than male stoats in captive trials [40], and Clapperton et al. [41] observed that captive female stoats did not interact with whole eggs or eat cracked egg contents. Griffiths [42] summarised the results of a series of stoat control operations and concluded that female stoats were less attracted than male stoats to egg lures. A more attractive lure than a whole egg or rabbit is needed, especially to trap female stoats. Not only are there sex differences in attractiveness to baits, but also individuality in prey preferences and behaviour can also mean that a one-size-fits-all approach to monitoring and trapping will not target all stoats adequately [43].

One approach to overcoming the limitations of food-based lures is to exploit natural odours used by the target species for communication. Stoats have a solitary lifestyle, defending intrasexual territories in the non-breeding season, and searching out mates in the breeding season [44,45,46]. They use scent from both anal and skin glands to mediate interactions between neighbours and strangers [47]. Scent marking is performed more by dominant than subordinate individuals but equally by males and females [47]. Preliminary work on mustelid scent lures focused on the highly odiferous sulphur-containing anal gland secretions [48,49]. While these odours were effective in attracting ferrets and stoats [50,51,52], synthetic lures made from the active ingredients were less attractive to stoats than to ferrets [53], and trials were discontinued. However, anal gland odours have been used successfully as trap lures to control another mustelid pest, the American mink (*Neovison vison* Schreber) [54,55,56]. Commercial lures containing unspecified polecat (*Mustela putorius*) and marten (*M. foina*) glandular secretions also increased the probability of detecting mustelids at camera-trap monitoring sites compared with no attractant [57]. 

Urine, faeces and other body odours are also potential sources of semiochemicals for mustelids [58]. Mustelids have skin glands that are potential sources of odours laid down as scent marks by body rubbing [59]. These odours may carry information about the sex and breeding status of the scent marker [60,61], making these materials potentially attractive odours for conspecifics. An invasive male stoat on Kapiti Island was caught in a trap in February 2011 containing the bedding material from an oestrous captive female stoat [62]. The scent marks are often associated with agonistic encounters and could act as a threat signal, indicating the close proximity of the scent marker [47]. However, it is to the advantage of subordinate individuals to ‘eavesdrop’ on the scent communication of dominant individuals or species to determine the freshness and identity of the scent, and thus assess the risk of encounter [32,35,63].

Stoats should therefore be expected to respond positively to the scents from glands of both male and female conspecifics and to approach the scent of larger predators as well as prey species. One source of a range of these potential lure compounds is the bedding materials found in dens of free-living or captive animals. The current study reports on a series of captive animal trials to assess the relative attractiveness to stoats of mustelid bedding material odours, supplemented by field trials to test their attractiveness relative to both rabbit- and egg-based lures.

## 2. Materials and Methods

### 2.1. Captive Animal Trials

#### 2.1.1. Animal Husbandry

The captive animal trials were conducted at the Johnstone Memorial Laboratory facilities of Lincoln University, Canterbury, New Zealand. Wild-caught stoats were housed individually in outdoor pens (3 m × 1.8 m × 2 m high) that contained foliage, wood and tunnels, with a nest box containing Dacron (polyethylene terephthalate) bedding material. The stoats were fed rabbit meat, hen eggs or 1-day-old chicks daily, and water was also available. Female stoats were not mated, so they remained in oestrus over spring and summer (September–February). Ferrets used as donors for odour test materials were housed in similar pens. 

#### 2.1.2. Test Design and Procedures

A series of trials was conducted on the captive animals between November 2013 and February 2017. In the first trial, 4 female and 4 male stoats were presented the urine and scats of an oestrous female stoat in November and December. In Trial 2, bedding odours of an oestrous female stoat were presented (a) to 5 female and 6 male stoats during the breeding season (November–February) and (b) to 3 females and 5 males in the non-breeding season (March–June). Different stoats were used in the two seasons. In Trial 3, 8 female and 6 male stoats were given the choice of bedding odours from an oestrous female stoat and those from a female ferret (collected in February, but the state of oestrus not confirmed). 

The stoats were tested individually in an outdoor pen (3 m × 6.1 m × 2.05 m in height). Bedding samples were collected from the donor animals’ cages and stored in zip-lock bags at room temperature until use. Single samples from individual donors were used in these pen trials. The control for all the trials was unscented Dacron. On the day of the trial, each sample was placed in a metal mesh tea ball. The researcher handling the lure materials wore different disposable gloves for each lure sample to guard against cross-contamination, and to minimize human scent, to which stoats produce agonistic responses [64]. The test materials were positioned on 45 cm metal stakes placed at least 1.5 m apart and from the nest box. The test animal could thus see and smell the lure material in the mesh balls but could not touch them. In multiple comparison tests, the control odour container was always positioned between the two test odours. Observations were recorded using a wide-angled overhead remotely operated camera.

The test animal was live-trapped from its home pen two nights before the trial and released into the pen. It was thus given two days/nights to explore the pen before the lures were introduced. Each trial was run over one 24 h period. After the trial, the stoat was returned to its home pen. The pen was water-blasted between trials. 

#### 2.1.3. Ethics Approval

Lincoln University Animal Ethics Committee approved these trials under applications 501, 535 and 630. 

### 2.2. Field Trials 

#### 2.2.1. Abel Tasman Trial

Stoat bedding lure was tested as part of a restoration project in Abel Tasman National Park. The bedding odour samples were prepared at the Lincoln captive animal facility and couriered to the field site. A trap line along the coastal section of the park was alternately lured with either oestrous female stoat bedding lure (presented in the mesh balls as described in the pen trials) or a dried rabbit meat block formulation (Erayz, Connovation NZ Ltd. Auckland, New Zealand. The trap stations were spaced 200 m apart and contained two DOC150 stoat traps within a wooden tunnel. The traps were serviced (checked, captures removed, lures replaced, and traps re-set) once a month between April and August 2014 (4 checks). Treatments were not rotated amongst the sites. In total, there were 66 stations treated with stoat bedding and 66 containing dried rabbit. Because most of the captured animals were well decayed by the time the traps were cleared, only species and not the sex of the captures was recorded; nor were rats identified to species but both *Rattus rattus* and *R. norvegicus* are likely to inhabit coastal forest [65]. 

#### 2.2.2. Rotoiti Trial

A field trial to compare the trapping success of oestrus stoat bedding and dried rabbit lures was conducted over two years in Nelson Lakes National Park as part of the Rotoiti Nature Recovery Project [66]. Nine trapping lines at Big Bush comprising a total of 308 traps placed 100 m apart were used from August 2016 to June 2018. For the first 8 months of the trial (August 2016 to mid-March 2017), traps were lured with either dried rabbit, or a combination of dried rabbit and oestrous female stoat bedding lure (prepared into the metal mesh dispensers as above except that cotton swabs were used instead of Dacron to collect the odorous material for 2 months in 2016). From mid-March 2017 to June 2018, the lures compared were dried rabbit versus oestrous stoat bedding lure on its own.

The lures were placed at the back of DOC200 and DOC250 single-set trap boxes. The treatments were alternated along the trap lines or between pairs of the same type of trap. The lines were checked, and the lures were refreshed monthly. Treatment position remained the same for the first year (August 2016–June 2017) and then reversed for the second year of the trial. Captured animals were recorded only by species. 

#### 2.2.3. Coromandel Trial

The Coromandel Kiwi Project team ran a 2-stage trial between August 2016 and March 2019 using their network of DOC200 traps in wooden tunnels in and around Coromandel township. Eleven trap lines were used, with tunnels spaced at 150–200 m intervals. In Stage 1 (6 months), half the traps were lured with male stoat bedding odour on a Dacron square (provided from Lincoln University) inside a paper tea filter bag plus either dried rabbit or a single hen egg. The alternate traps contained either the dried rabbit or hen egg lure, depending on the monthly lure regime and varied among lines. In Stage 2 (2 years), the male stoat bedding lure was tested on its own against either dried rabbit or a hen egg. There were 78 bedding lure treatment trap sites and 85 food-lured trap sites. Treatments were not rotated amongst the sites. Each trap was checked monthly as above by volunteers and with occasional additional opportunistic checks when a member of the public reported that a tunnel contained a capture. 

### 2.3. Data Analysis

The video recordings for the captive trials were mostly reviewed by co-authors T.S., T.A. and M.S., who did not know which treatment was which. The total amount of time each stoat spent touching the lure dispensers over a 24 h period was recorded. The results were analysed using GLMM with a negative binomial link function to avoid overdispersion. Fixed effects in the model were lure type and sex of the test animal with test animal ID as a random effect. Where significant differences were detected, pairwise comparisons of means were undertaken using the Tukey-adjusted *p*-value method, performed on the log scale.

Comparisons of catch rates of animals trapped in the field on different lure types were analysed using a 2-sample test for equality of proportions without continuity correction given the large sample sizes. Analysis was performed using trap site nights with a double set (i.e., two traps in one box) recorded as a two for each night deployed in the field (Abel Tasman) and a single set trap recorded as one for each night (Rotoiti and Coromandel). 

All data analysis was conducted using the statistical package R (version 4.0.2) using packages lme4 (version 1.1–27.1) and emmeans (version 1.6.2-1). 

## 3. Results

### 3.1. Captive Animal Trials

The stoats would respond to the odour stations by approaching the stake, typically sniffing at the base and then standing on their back legs to sniff directly with the nose touching the lure dispenser.

#### 3.1.1. Oestrous Urine/Faeces

In this trial run in spring and early summer (breeding season), there was a significant difference (χ^2^ = 10.98; *p* < 0.001) in the amount of time stoats spent investigating the urine and scats odours (13.85 ± 3.0 s) compared with the blank control (6.33 ± 1.5 s). The difference between males and females was close to being significant (χ^2^ = 3.48; *p* = 0.06). The response times ranged from 1 to 54 s for the oestrous lure compared with 1 to 19 s for the control.

#### 3.1.2. Oestrous Female

Table 1 gives the mean time spent by stoats interacting with the oestrous bedding lures in the breeding and non-breeding season. There was a significant interaction between lure type and sex (χ^2^ = 14.36; *p* < 0.001), with male stoats responding more than female stoats to the oestrous lure. Both male and female stoats spent significantly more time interacting with the treatment than with the control (both *p* ≤ 0.001). The response of male stoats to oestrous bedding odours tested in the breeding season ranged from 37 to 271 s and for female stoats 11 to 243 s. 

In the non-breeding season, there was still a significant interaction between lure type and sex (χ^2^ = 32.01; *p* < 0.001), with male stoats responding more than female stoats to the oestrous lure (Table 1). Both were again significant when compared with the control (both *p* ≤ 0.001). The response of male stoats to oestrous bedding odours tested in the non-breeding season ranged from 61 to 444 s and for females from 39 to 230 s.

#### 3.1.3. Oestrous Female Stoat vs. Female Ferret 

As above, there was a significant interaction between lure type and sex (χ^2^ = 29.10; *p* < 0.001). This time female stoats responded significantly more than male stoats to both the oestrous stoat and the ferret lure (Table 2). 

All the contrasts were significant. Female stoats responded significantly more to stoat bedding than either ferret bedding (*p* < 0.001) or the control (*p* < 0.001), and significantly more to ferret bedding than the control (*p* < 0.001). Male stoats responded significantly more to stoat bedding than either ferret bedding (*p* < 0.001) or the control (*p* < 0.001), and significantly more to ferret bedding than the control (*p* < 0.001). 

The response of female stoats to oestrous bedding ranged from 18 to 184 s, and for ferret bedding, from 12 to 126 s. For male stoats, the response ranged from 0 to 120 for oestrous bedding and from 0 to 59 s for ferret bedding (note 1 male stoat did not respond to any of the lures).

### 3.2. Field Trials

#### 3.2.1. Abel Tasman Trial

The numbers of stoats and rats caught during the Abel Tasman trial are summarised in Table 3. The catch rate of stoats on the oestrous female stoat bedding lure was more than double that of the dried rabbit lure but the difference was not significant (χ^2^ = 2.58, *df* = 1, *p* = 0.11). Two of the stoats caught with dried rabbit were caught at the same site during the same monthly trapping period. Significantly more rats were caught on dried rabbit than the oestrous bedding lure (χ^2^ = 4.25, *df* = 1, *p* < 0.05) 

#### 3.2.2. Rotoiti Trial

The captures recorded during the trial at Nelson Lakes are summarised in Table 4. In the first stage, similar numbers of stoats were caught on dried rabbit alone and on dried rabbit + oestrous stoat bedding lure (χ^2^ = 0.16, *df* = 1, *p* = 0.69). In the second stage, testing the bedding lure on its own versus the dried rabbit lure, again there was no significant difference in the number of stoats caught (χ^2^ = 0.33, *df* = 1, *p* = 0.56).

The addition of stoat bedding odour to the traps did not significantly affect capture rates of rats in Stage 1 (χ^2^ = 0.27, *df* = 1, *p* = 0.61). However, stoat bedding lure on its own in Stage 2 was significantly less attractive to rats than the dried rabbit lure (χ^2^ = 10.86, *df* = 1, *p* = 0.001).

#### 3.2.3. Coromandel Trial

The trial results of male stoat bedding lure against dried rabbit and egg lures in Coromandel are summarised in Table 5. In Stage 1, adding the male stoat bedding odour did not significantly affect the number of stoats caught with dried rabbit lure (χ^2^ = 0.57, *df* = 1, *p* = 0.45). The number of stoats caught on egg lure in Stage 1 also did not vary significantly with the male stoat bedding lure or without it (χ^2^ = 0.44, *df* = 1, *p* = 0.51). In Stage 2, again there was no significant difference between the number of stoats caught on the male stoat bedding lure or dried rabbit (χ^2^ = 2.78, *df* = 1, *p* = 0.10), nor between the male stoat bedding lure and egg (χ^2^ = 0.51, *df* = 1, *p* = 0.47). 

Two ferrets were caught, one in Stage 1 on the dried rabbit/stoat odour lure and the other in Stage 2 on the stoat lure. Only three weasels (*Mustela nivalis vulgaris* Erxleben) were caught in Stage 1 and 22 in Stage 2 (Table 5). They did not show any preference for or avoidance of the stoat lure treatments.

Rats were caught on the male stoat bedding lure both when presented in Stage 1 together with the rabbit or egg lure and on their own in Stage 2 (Table 5). The addition of the stoat lure did not affect the efficacy of dried rabbit (χ^2^ = 0.01, *df* = 1, *p* = 0.98) but significantly increased the number of rats caught on the egg lure (χ^2^ = 12.18, *df* = 1, *p* < 0.001). When presented on its own in Stage 2, the stoat lure caught significantly fewer rats than the dried rabbit lure (χ^2^ = 11.48, *df* = 1, *p* < 0.001) but significantly outperformed the egg lure (χ^2^ = 7.72, *df* = 1, *p* < 0.01).

## 4. Discussion

In both captive and field trials, the results described here have repeatedly demonstrated the potential for bedding odours as lures for stoats. The trials on captive animals showed that both male and female stoats would spend time investigating the scent of conspecifics, in both the breeding season and the non-breeding season. Because the female scent used was always from animals in oestrus, it is possible that the male stoats were responding to an oestrous signal, even during the non-breeding season. This would explain the higher responsiveness of the males compared with the females. However, that the female stoats also responded to the female bedding odours and that male bedding odours were effective in the Coromandel field trial indicate that the stoats were more likely responding to a general mustelid scent. The attractiveness of the bedding lure to the female stoats, although not as high as to the males, is a positive result for the control of the traditionally hard to trap females. The captive stoats also responded to the bedding odour of a female ferret, as was found by Garvey et al. [35].

The field trial results indicate that bedding lures can be as effective as a dried rabbit lure or hen egg for attracting stoats to kill traps. This supports the findings of Garvey et al. [32], who showed that ferret bedding odours have potential as a lure for monitoring mustelid populations via camera traps or other monitoring devices. While we did not include a blank control in the field trials, we can confidently assume that the lack of difference between the treatments was not because of a lack of need for a lure. Previous studies have shown differences in catch rates on different lures [67,68]. The anal gland odour of stoats has also been shown to be an effective stoat attractant against blank controls in tracking tunnels [52]. 

Our results indicate that bedding lures also have the potential to be used in combination with a food odour. The combination of lures is not likely to provide conflicting messages—stoats use body rubbing to scent mark food caches [47], and ferrets likewise mark food sites by chin rubbing [69]. Garvey et al. [32] reported that stoats interacted with rabbit baits combined with ferret body odour more than with rabbit meat alone. Mink have also been caught in traps treated with both fish bait and mink anal gland scent [70].

Mustelid bedding odours also have potential as multi-species mustelid lures. This is supported by (a) the positive responses of the captive female stoats to the ferret bedding odours, and (b) the fact that both weasels and ferrets (but not cats, *Felis catus*) were caught on the stoat bedding lures in the field trials. These responses support the results of trials by Garvey et al. [32,35] and field trials reported by Harrington et al. [71] which indicated that, although they may be most responsive to their own odour [72], mustelid species are attracted to each other’s odours. This is consistent with the current view that carnivores use interspecific scent communication [73].

Although there were higher capture rates of rats on dried rabbit than stoat bedding odours in the field trials, the fact that over one-third of rat captures were in traps containing stoat bedding lure indicates that this material is not highly repellent to rats. The results of the Coromandel trial indicate that it may even be a more effective lure than whole hen egg for rats. This is consistent with the results of laboratory trials that have shown that rats show risk assessment and investigatory behaviours towards mustelid body odours but not fear responses [74,75,76]. In addition, ferret body odours did not reduce visitation rates of rats to scent stations in a field trial reported by Garvey et al. [32].

We need more in-depth trials measuring lure efficacy over shorter time frames than one month to determine how long the bedding odours remain attractive to the target animals. Field or laboratory assessment of age and sex of captured stoats would also add additional information on the breadth of lure attractiveness. More stable formulations may be required, for example, incorporating the odours into slow-release mechanisms [51,52,53]. Alternatively, identification and synthesis of the active components may be required for adequate lure availability, and initial investigations have begun (A. Twidle, Plant & Food Research Ltd., Lincoln, New Zealand, unpublished data).

## 5. Conclusions

Results from both captive and field trials demonstrate the potential of body odours as trap attractants for mustelid control. Stoat bedding odours and urine/faeces were attractive to both male and female stoats, and both male and female odours were effective attractants. Further work may include a comparison of the attractiveness of bedding and anal gland odours, lure formulation testing and the development of a synthetic lure based on naturally occurring compounds, to allow upscaling of lure production. Eradicating stoats from New Zealand will be a significant challenge. Any single technique is likely to leave some survivors that avoid that technique, and multiple methods will be required. We believe that social lures are likely to be an important addition to the toolbox and will be particularly useful in situations where food is abundant (and food-based lures are therefore less effective) and/or where the density of conspecifics is low, and survivors are seeking mating opportunities.

## Figures and Tables

**Table 1:** Mean (±SE) time spent by stoats interacting with the bedding odour of an oestrous female stoat during the breeding and non-breeding seasons. The odours were collected on Dacron bedding material and compared with a blank piece of Dacron (control).

Interaction Time (s)	*n*	Oestrous Female		Control	
		Mean	SE	Mean	SE
Breeding season					
Females	5	57.5	27.0	10.1	4.9
Males	6	82.8	35.4	33.1	14.3
Non-breeding season					
Females	3	87.8	43.2	38.4	19.1
Males	5	119.6	45.5	16.6	6.5

**Table 2:** Mean (±SE) time spent by stoats interacting with the bedding odour of an oestrous female stoat, and a female ferret during spring, summer and autumn. The odours were collected on Dacron bedding material and compared with a blank piece of Dacron (control).

Interaction Time (s)	*n*	Stoat		Ferret		Control	
		Mean	SE	Mean	SE	Mean	SE
Females	6	66.9	1.1	48.0	0.8	13.8	0.2
Males	8	19.4	0.5	13.8	0.3	11.9	0.2

**Table 3:** Captures of stoats and rats on dried rabbit meat or female stoat bedding lure during the field trials at Abel Tasman, April–August 2014.

	Rabbit	Female Odour
Trap nights ^1^	6775	6775
Sites with stoat captures ^2^	5	13
Total no. stoats caught	6	13
Sites with rat captures ^2^	99	77
Total no. rats caught	115	86

^1^ Traps were double sets. ^2^ Excluding double captures in one trapping period.

**Table 4:** Captures of stoats, other predators and non-target species on dried rabbit meat, with and without female stoat bedding lure during the field trials at Lake Rotoiti, 2016–2018.

Stage 1 August 2016–Mid March 2017		
	Rabbit	Rabbit + Female Odour
Trap nights	34,958	34,958
Stoat	26	29
Weasel	0	1
Ferret	0	0
Cat	2	0
Rat	71	65
Mouse	1	3
Possum	0	0
Hedgehog	22	38
Rabbit	12	9
Bird	0	0
**Stage 2 Mid March 2017–June 2018**		
	**Rabbit**	**Female Odour**
Trap nights	86,548	86,548
Stoat	40	35
Weasel	10	5
Ferret	2	2
Cat	10	0
Rat	196	136
Mouse	3	0
Possum	2	0
Hedgehog	77	41
Rabbit	6	22
Bird	1	0

**Table 5:** Captures of stoats, other predators and non-target species on dried rabbit meat, hen egg and/or male stoat bedding lure during the field trials at Coromandel, 2016–2019. Stage 1 tested the food lure against a combination of scent lure and food lure. In Stage 2, the scent lure was tested on its own against the food lure. The two food lures were used during different monthly trapping periods on the various trapping lines. Total trap nights are shown for each lure treatment.

Stage 1 August 2016–February 2017				
	Rabbit	Rabbit + Male Odour	Egg	Egg + Male Odour
Trap nights	7395	6772	6883	6421
Stoat	11	7	6	8
Weasel	1	0	0	2
Ferret	0	1	0	0
Rat	68	62	25	53
Hedgehog	0	0	4	2
Rabbit	0	0	0	0
**Stage 2 March 2017–March 2019**				
	**Rabbit**	**Male Odour**	**Egg**	**Male Odour**
Trap nights	19,562	18,179	41,943	39,088
Stoat	26	14	43	34
Weasel	2	3	9	8
Ferret	0	0	0	1
Rat	209	134	203	245
Hedgehog	3	1	8	2
Rabbit	1	0	0	0

## Data Availability

The data presented in this study are available on request from the corresponding author.
